# Bridging the genotype–phenotype gap in 3D

**DOI:** 10.1093/jxb/erw264

**Published:** 2016-08-03

**Authors:** Paul C. Struik

**Affiliations:** Centre for Crop Systems Analysis, Plant Sciences Group, Wageningen University and Research, Droevendaalsesteeg 1, 6708 PB Wageningen, the Netherlands

**Keywords:** 3D modelling, allometric relationships, functional–structural plant model, gene-based modelling, plant architecture, three-dimensional canopy structure, virtual plants

**Three-dimensional architectural models have become important tools for studying physiological relationships between form and function in crop stands. Incorporating genetic variation in architectural traits would allow the use of such models for model-based, marker-assisted breeding. Perez *et al.* (pages 4507–4521 in this issue) applied mixed-effect models on allometric relationships to account for genetic variability in the 3D architecture of oil palm, demonstrating that there is a trade-off between model accuracy and ease of defining parameters for the 3D reconstruction.**

Crops are highly plastic: they respond to environmental cues and management interventions by changing morphological and architectural traits and adjusting their physiological behaviour. In recent years much progress has been made in developing dynamic functional–structural plant models (FSPMs) that combine the representation of 3D plant and canopy structure over time with specific (changes in) physiological behaviour and quantify complex interactions between architecture and physiological processes (Vos *et al.*, 2010).

The architectural part of a functional–structural plant or crop model describes the types of organs that are initiated, the coordination in the dynamics of organ growth and development, and geometric variables (such as leaf angle, azimuth, torsion and curvature). The physiological part could include, for example, modules to describe light capture, localized photosynthesis, and carbon and nitrogen allocation.

In many crops, growers actively manipulate plant structure and the principles behind these impacts can also be modelled (e.g. impact of pruning roses on bud break). In crop science, leaf area (and its dynamics) is an obvious architectural trait to model as it is directly coupled to light interception and photosynthesis. At any time during the crop cycle, the number of branches formed, their position and the position and area of their leaves determine the total leaf area of the plant, the spatial distribution of leaf area in the canopy and, thus, the amounts of light absorbed and photosynthates produced (Evers *et al.*, 2011).

## Genotype-specific modelling to support breeding

The idea that process-based physiological models of crop growth could support breeding was suggested decades ago (e.g. Loomis *et al.*, 1979). Such models require environmental (e.g. weather variables) and physiological inputs. The latter model-input traits can be used as genotype-specific model parameters to characterize genotypic differences; in fact, it can be surmised that they are, at least partly, under genetic control (Yin *et al.*, 2016).

Breeders can use models to: (i) identify the main yield-determining traits and their most significant markers, (ii) define the best selective environments for maximizing the progress of selection, (iii) optimize single-trait values, (iv) design ideotypes, and (v) assist multi-location testing (Gu *et al.*, 2014; Yin *et al.*, 2016).

However, Jackson *et al.* (1996) warned that models may not identify those traits for which gain via breeding is easiest. Koornneef and Stam (2001) expressed their concerns that such modelling approaches ignore the complex inheritance of the model-input traits, for example by ignoring the possible existence of constraints, feedback mechanisms and correlations among traits.

## Towards gene-based functional–structural plant models

With the rapid development of ‘omics’ sciences and technologies, FSPMs may also play a role in evaluating genetic traits across environments for crop performance. The ultimate goal of such efforts could be the construction of architectural ideotypes, representing optimal ranges for individual architectural traits that would contribute to achieving maximum yield (Xu and Buck-Sorlin, 2016). Such traits may include many different architectural components, such as branching intensity, rate of leaf appearance, leaf blade angle, and mechanical properties of the stem, petiole and rachis. First attempts to include modules for genetics in FSPMs have already been made. Luquet *et al.* (2012) proposed an FSPM for rice in which its growth rate was parameterized with different genotype effects. Xu and Buck-Sorlin (2016) introduced a genotype–phenotype module coupling quantitative genetic information on plant height with the morphogenetic rules leading to this complex trait in an FSPM for rice. However, given the large number of traits involved, their strong interactions and their strong responses to environmental factors, making a gene-based FSPM is very complex. Even including genotype × environment interactions in an FSPM is a huge task.

## The case of inter- and intra-progeny variability in oil palm

Perez *et al.* (2016) investigated variability in 3D architecture for oil palm (*Elaeis guineensis*). It is a suitable model plant as it has a simple architectural topology, characterized by a mono-axial shoot producing phytomers in a regular succession and arranged with radial symmetry, following Corner’s architectural model (Box 1). Individual leaves, however, are complex as they consist of many leaflets, varying in shape and size.

**Box 1. The architectural model of the oil palm**

(a) Oil palms follow Corner’s architectural model (Hallé *et al.*, 1978), characterized by a unique apical bud producing phytomers, each bearing a leaf with either a male or a female inflorescence at its axil. Expanded leaves are indicated by L^1^ to L ^*n*^ , with the number in each case indicating the rank (rank 1 being the youngest leaf with fully unfolded leaflets) down to lower and older leaves, which are generally pruned in order to ease the harvest of bunches, i.e. when they reach a rank between L^32^ and L^40^. Unexpanded leaves packed in the bud are indicated by L^−1^ to L^−*n*^. Oil palms produce about two fronds per month and therefore the rank of a phytomer is increased by 1 every two weeks. (b) Phenological stages of leaves and inflorescences: leaves are initiated more than two years before emerging from the apical bud. Inflorescences are initiated a little time after and their sex determination occurs at phytomer ranks L^−25^ to L^−15^. Inflorescence abortion occurs mainly before anthesis at ranks L^8^ to L^12^. See Corley and Tinker (2003) for details of oil palm phenology; and the phytomer modelling concept by Fan *et al.* (2015), with similar palm representation; supplement to Fan *et al.* (2015): *Geoscientific Model Development* (Fan Y, Roupsard O, Bernoux M, Le Maire G, Panferov O, Kotowska MM, Knohl A. 2015. A sub-canopy structure for simulating oil palm in the Community Land Model (CLM-Palm): phenology, allocation and yield. Geoscientific Model Development 8, 3785–3800). © Fan *et al.*, 2015; Creative Commons Attribution 3.0 Licence. Courtesy of Dr Jean Dauzat.

**Figure:**
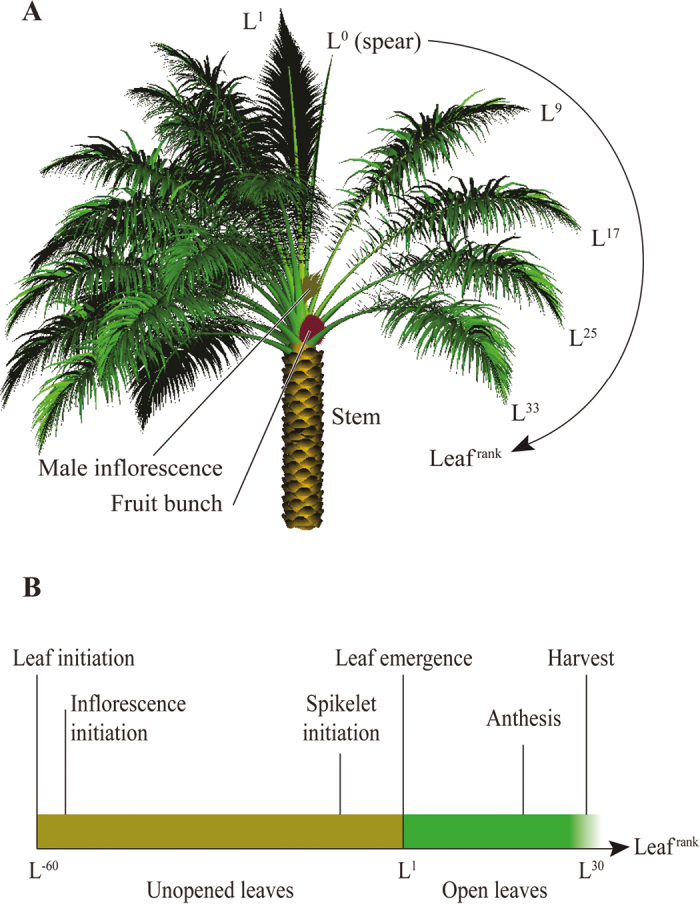

As for all other crop species, this topology works in two ways: it determines the interception and use of solar radiation and it affects the microclimate within the canopy. Making use of architectural models requires plant developmental models to be combined with biophysical models (e.g. energy balance models) and physiological models (e.g. photosynthesis models).

It is difficult to assess architecture precisely. Therefore, allometric relationships are required that reflect the morphological relationships between plant components at different scales of organization and model geometric gradients of plant components as a function of temporal and spatial variables. When such relationships are combined with clever sampling strategies, they can be used to reconstruct the 3D architecture of a single plant from the individual organ all the way up to the entire crop stand. Obviously, models of the 3D architecture of an oil palm plant should be made dynamic as many architectural traits show clear development over time.

Perez *et al.* (2016) estimated both the inter- and the intra-genotypic variability of architectural traits and allometric relationships in order to break down the variability into causal components applying mixed-effect models, resulting in genotypic values, heritabilities of traits and genetic correlations between variables.

At plant level, the authors introduced number of leaves emerged after planting date and leaf rank to account for morphogenetic gradients of leaves in the crown: rachis length was estimated based on number of leaves emerged and rachis declination was modelled as a function of leaf rank. At leaf level, the relative metric position on the rachis was used to describe the evolution of the rachis segment angles, azimuth and twist. Leaflet attributes were linked to their relative position along the rachis. Modelling leaflet shape was based on the relative position of the leaflet midrib.

The predictions per progeny based on direct data were generally good, but variables simulated from a combination of various allometric relationships gave greater discrepancies between observed and predicted values. Predictions of morphogenetic gradients worked well. The 3D mock-ups of each progeny studied showed that the model was capable of simulating the architectural genotypic characteristics well. The authors also demonstrated that there was a trade-off between model accuracy and ease of defining parameters for the 3D construction.

## Significance and implications

Using allometry to analyse genotypic variability proved to be very useful, and the mixed-effect model which was applied worked very well. The significance of the paper is that it provides a detailed analysis of genotypic variability in architectural traits of oil palm, which is very useful given the complexity of oil palm breeding. However, it should be noted that the architecture of the oil palm plant is relatively simple; it is the individual leaf which is complex.

The authors want to use their findings to carry out sensitivity analyses and to couple the architectural model to a radiative balance model in order to identify key architectural traits involved in light interception. Similar research strategies can be applied to crops with a more complicated architecture, for example to unravel branching patterns (Evers *et al.*, 2011) by coupling the physiological aspects of bud break and branch formation to the allometric structure of leaf area along the main stem in association with photosynthesis of particular leaves based on penetration, scattering and distribution of light (Evers *et al.*, 2010).

Moreover, by including modules for photosynthetic capacity, resource allocation in the canopy profile and light distribution, photosynthesis can be scaled up from leaf to canopy in a dynamic way taking account of phenology and different levels of localized resources, such as light and nitrogen (e.g. see Evers *et al.*, 2010, in wheat).

## Perspectives

Yin & Struik (2016) indicated that different temporal, spatial and structural scales are required to describe different components, pathways and processes in a model. Chew *et al.* (2014) described a multiscale model for Arabidopsis that integrated gene dynamics, carbon partitioning, organ architecture and development responses to endogenous and environmental signals. Such approaches using coupled models can even inform how and where recalcitrant genetic phenomena (G × E interactions, epistasis, pleiotropy) come about (Yin & Struik, 2016), avoiding the pitfalls mentioned by Koornneef and Stam (2001). Ultimately, such models will allow virtual ideotyping and *in silico* assessment of crop performance after genetic fine-tuning under defined environmental scenarios (see Box 2).

**Box 2. Improving oil palm plant and canopy structure**

Diagram of methodology for ideotyping and *in silico* breeding to improve oil palm plant and canopy structure. FSPM, functional–structural plant model; QTL(s), quantitative trait locus (loci).

**Figure:**
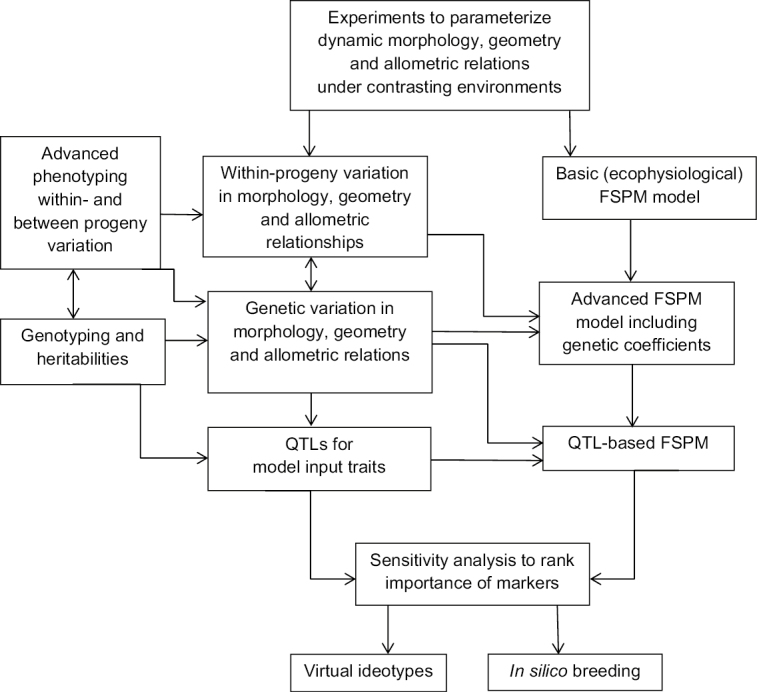

Xu and Buck-Sorlin (2016) already gave it a try: for the first time they provided an extension of an FSPM for rice with a module for genetics, which constitutes a genotype–phenotype model coupling quantitative genetic information of the phenotypic trait plant height with the morphogenetic rules leading to this composite trait. They also provided a virtual breeding model enabling the virtual reproduction of quantitative genetic information and the generation of a new simulated mapping population, in both its phenotypic and genotypic form.

A lot of ground still needs to be covered in working out the details, but this is a fascinating and rapidly developing discipline which will contribute greatly to unravelling the phenotype–genotype gap, and more specifically the 3D aspects of that gap.
